# Supplementary material for: A model of colour appearance based on efficient coding of natural images
Source: PLoS Comput Biol. 2023 Jun 15;19(6):e1011117. doi: 10.1371/journal.pcbi.1011117 (PMC10270630; doi:10.1371/journal.pcbi.1011117)
Supplement: S1 Appendix — (PDF) [file pcbi.1011117.s001.pdf]

S1 appendix to "*A model of colour appearance based on efficient coding of natural images*" by Jolyon Troscianko & Daniel Osorio

Key	
	Predicts effect and relevant controls
	Predicts effect, but not controls, or only partially predicts effect
	Does not correctly predict effect

Phenomenon Family	Phenomenon	Description	*Source*	DoG (non-oriented) Model		Gabor (oriented) Model	
Crispening Effect	The crispening effect causes perceived contrasts to be greater when the grey levels are nearer those of the background. The effect was modelled by Whittle [1], and subsequent work suggests the dipper effect [16] and divisive gain explains the effect [17]. Here we use Whittle's 1992 data to determine the dynamic range of human luminance vision.						
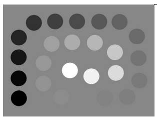	Grey background	Human subjects adjusted grey targets in equal-contrast steps on a grey background	*Generated from Whittle's Data [1]*	DoG fit, DR=15, R^2=0.994	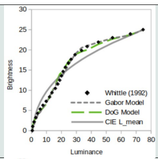	Gabor fit, DR=3.75, R^2=0.995	Both models outperform CIE L (R^2=0.944)
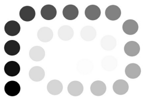	White background	As above with white background		DoG fit when using the above DR R^2 = 0.946	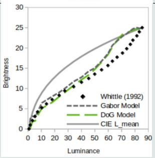	Gabor fit when using the above DR, R^2 = 0.935	Both models outperform CIE L (R^2=0.746)
Contrast sensitivity	The ability of humans and other animals to perceive contrasts is dependent on the spatial frequency of those contrasts. Contrast sensitivity functions describe the contrast a of a sinwave that is detectable at different spatial frequencies. A related phenomenon is contrast constancy, where suprathreshold contrasts appear to be uniform irrespective of spatial frequency.						
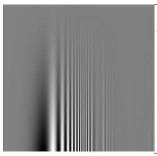	Contrast sensitivity functions	Sinewaves are generated with specific Michelson contrasts to ensure the model only permits detectable contrasts.	*Generated*	Removes sub-threshold contrasts, matching CSF	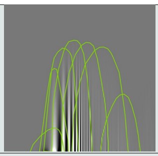		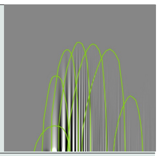
	Contrast constancy	Suprathreshold sinewaves of different spatial frequencies should have equal amplitudes.		Suprathreshold contrast constancy is enhanced by saturation thresholds preventing multiplicative gain effects.			
Brightness illusions	This family of illusions causes grey targets to differ in perceived brightness dependent on the arrangement of (typically high contrast) surrounds. Some of these illusions, such as simultaneous contrast and Mach bands have traditionally been attributed to centre-surround antagonism [18]. However the White illusions create the opposite effect, and have variously been attributed to oriented filtering with normalisation [3, 19], T-junctions [e.g. 20], Gestalt/grouping/anchoring based mechanisms [5]. A further set of illusions have been attributed to 3D surface and lighting based inferences [see 20], or atmospheric-based inferences [see 20].						
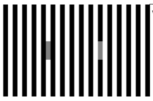	White's bars	A grey bar flanked by black appears darker than the same grey flanked by white	*Adapted from [2] & [3]*		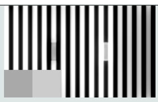		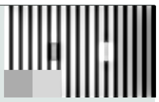
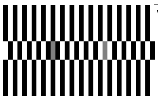	White's offset bars	As above with offset surrounds			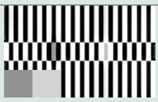		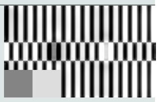
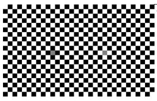	White's checkerboard	A grey square flanked by black squares appears darker than the same grey flanked by white squares			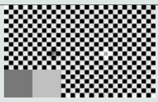		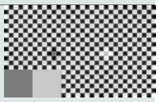
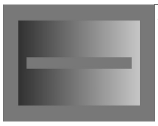	Simultaneous brightness contrast	The central grey bar is a uniform grey value, but the gradient in the background creates a powerful inverse luminance gradient in the bar. This is typically explained by centre-surround antagonism.	*Generated*	Both models create an inverse gradient, though the Gabor model's is more linear across the entire bar length.	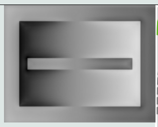	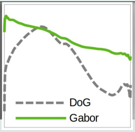	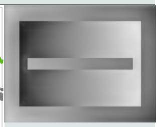

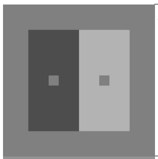	Simultaneous brightness contrast	A grey square surrounded by black appears darker than the same grey surrounded by white.	*Adapted from [3]*		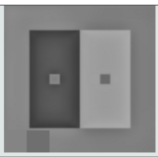		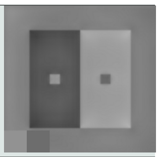
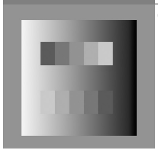	Chevreul staircase	The steps in a sequence of grey levels from light to dark appear flat/homogeneous on a contrasting gradient, but when viewed against a matching gradient each step appears to have a strong internal gradient.		The internal gradients are much stronger in the lower rather than upper staircase	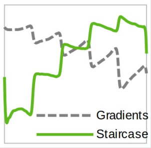	The internal gradients are much stronger in the lower rather than upper staircase	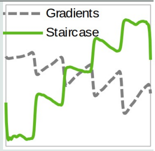
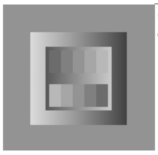	Chevreul staircase control	Geier & Hudák (2011) find that the illusion persists when a counter-gradient surround is placed around the illusion, and suggest that traditional centre-surround antagonism cannot explain the effect.	*Adapted from [4]*	As above, though the effect is not as powerful	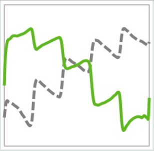	As above, though the effect is not as powerful	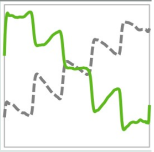
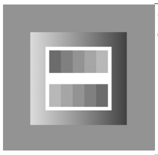	Chevreul staircase control	As above, however a white surround is found to eliminate the internal gradients of the staircases.		Still retains fairly clear internal gradients, although they are less powerful than above	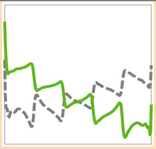	Still retains fairly clear internal gradients, although they are less powerful than above	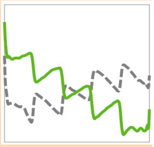
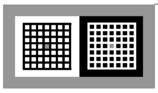	Dungeon illusion	A light grid causes a grey rectangle to appear lighter than the same grey surrounded by a dark grid.	*[5]*		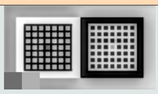		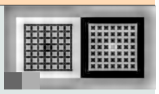
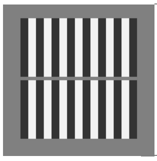	Grating induction	Illusory checkerboard patterns are created in a horizontal grey bar placed over a vertical grating.	*Adapted from [3]*		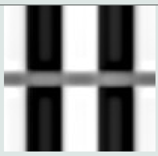		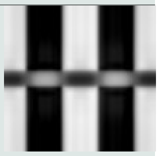
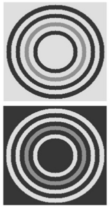	Hong-Shevell illusion	Circular variant of White's bar illusion. The grey ring neighbouring white rings appears lighter, and the same grey neighbouring dark rings appears darker.	*From [3]*		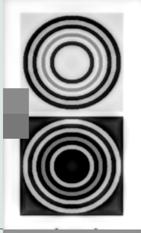		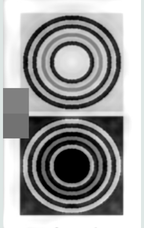
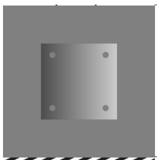	Luminance illusion	Simultaneous brightness illusion that uses a background gradient.			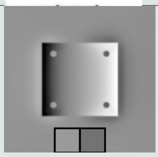		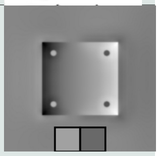
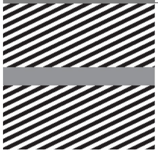	Poggendorff illusion	Illusory stripes are created in a grey bar placed over a diagonal grating.		Illusory stripes don't span the entire height of the bar	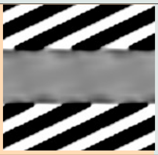		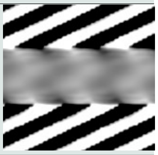
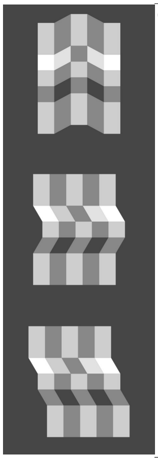	Corrugated plaid	The perceived brightness of identical grey patches on a checkerboard can be altered by various 3D and shading manipulations. The controls demonstrate how 3D-inference does not explain the effect [20].	*Figures from [20]*	Correctly predicts the direction and approximate magnitude of the effect. i.e. the effect is most powerful in the lower two versions with a parallelogram (rather than square) tile. Effect is 31% more powerful in the middle, and 34% more powerful in the lower version compared to the top.	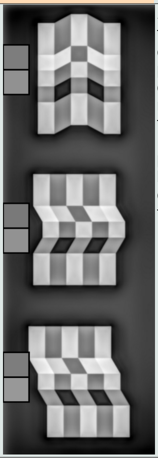	Same as DoG to the left, although even more powerful. The effect is 296% more powerful in the middle, and 147% more powerful in the lower version compared to the top.	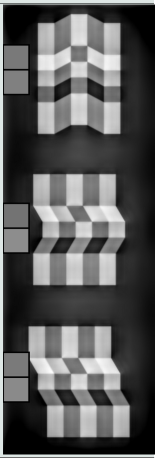

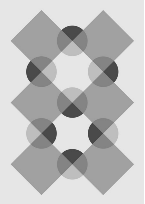	Haze illusion	Dark, high contrast surrounds increase perceived brightness of the lower tile. Adelson attributes the effect to perceived atmospheric differences between the tiles.		Lower tile 11% brighter than upper tile	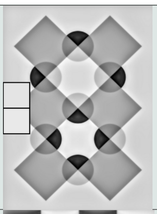	Lower tile 21% brighter than upper tile	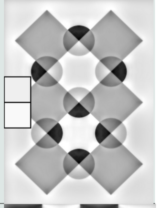
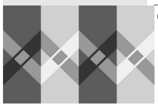	Crisscross illusion	A patterned grey target surrounded by a light background appears darker than the same grey with dark surrounds. Note this is the opposite effect of White's illusions, and is similar to simultaneous contrast.			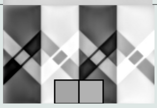		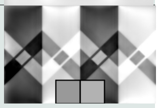
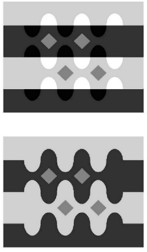	Snake illusion	Similar to the crisscross illusion above, however a control shows how the effect can be negated by removing "atmospheric" bands.		Brightness illusion in the upper version with haze layer is more powerful than the lower (control)	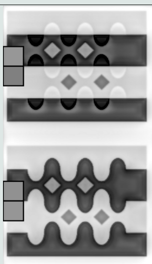	Same as DoG (left), with an even larger difference between upper and lower	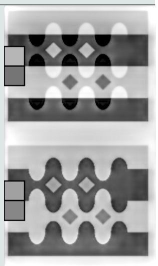
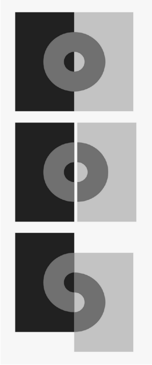	Koffka rings	An intact grey ring appears uniform when viewed against a split light/dark surround. However, when the ring is split into two halves and separated slightly the two sides have a strong brightness difference. Offsetting the rings has a similar effect.		The separated ring (centre) has a contrast between left and right sides 51% higher than the intact ring, and the offset ring (lower) has a contrast 8% higher than the intact ring. A lower dynamic range can eliminate all internal contrast in the intact ring.	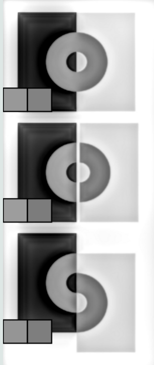	Same as DoG (left), separated ring is 66% higher contrast and offset ring is 13% higher contrast than the intact ring. Likewise, the effect is enhanced with a lower dynamic range.	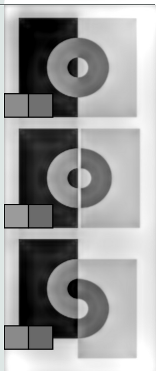
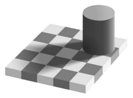	Adelson checker shadow illusion	The shadow cast onto the checkerboard causes the shaded square to appear brighter than a square with the same grey level outside of the shadow.	*Adelson (1995). Retrieved from wikimedia.*		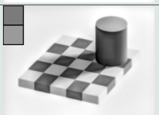		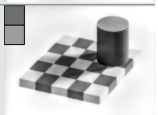
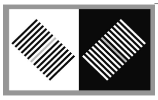	Reverse contrast illusion	The grey diagonal bar surrounded by black bars and white background appears brighter, and the opposite is true for an inverted example.	*[5]*		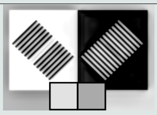		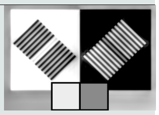
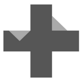	Benary cross illusion	The triangle cutting into the arm of the cross appears brighter than the triangle that spans between two arms.			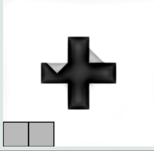		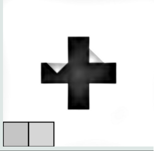
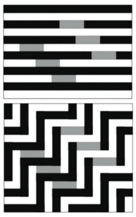	Wedding cake illusion	Variant of White's bar illusion with zigzag background	*[6]*		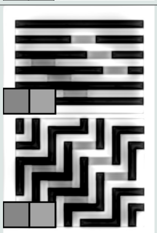		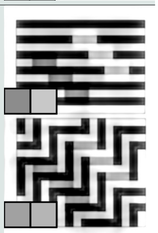
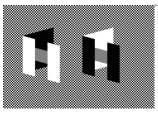	Figure 2	Zaidi [7] provide a detailed study of the magnitude of various brightness induction effects. They use t-junction theory to model the predicted outcomes.<br><br>The study details the experimental viewing conditions, allowing for accurate representation and model testing. Although the authors do quantify the magnitude of the effects (i.e. the brightness adjustments required to nullify the illusion) only two subjects were used, meaning it is impossible to quantify	*Generated following [7], matching luminance and viewing angle precisely*	Marginal effect in wrong direction	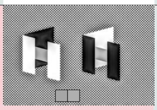		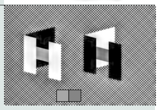
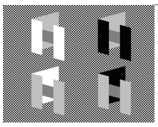	Figure 3				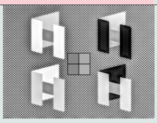		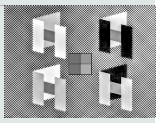

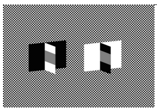	Figure 4	variance and therefore the magnitude of differences. Moreover, we note that the effects our Gabor model fails to predict well are also effects that are only marginally visible to us.		Marginal effect in correct direction	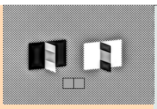		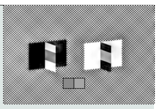
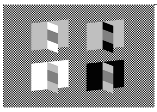	Figure 5				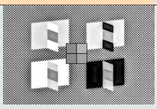		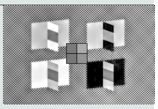
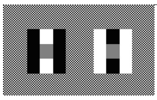	Figure 6			Marginal effect in wrong direction	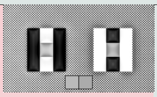		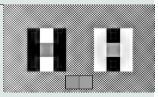
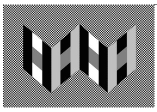	Figure 8				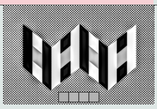		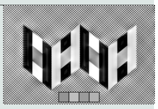
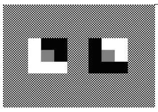	Figure 9			Marginal effect in correct direction	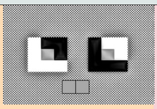	Predicts no difference	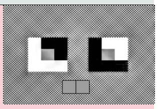
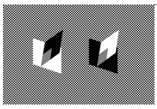	Figure 10			Marginal effect in wrong direction, although the illusion itself is weak	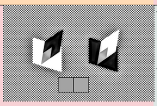	We do not perceive a strong illusory effect from this stimulus	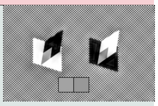
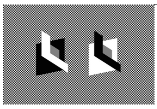	Figure 11			Marginal effect in wrong direction, although the illusion itself is weak	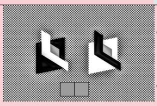	Marginal effect in wrong direction, although the illusion itself is weak	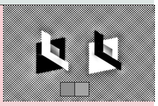
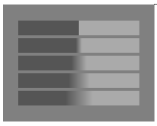	Mach bands	Mach bands are the perceived light and dark stripes created where a ramp of grey meets a flat grey. Mach bands are traditionally explained by centre-surround antagonism, but other theories have been used to explain their presence or absence [see Kingdom (2014)].	*Generated following [8]*	Predicts the Mach band effect will be most powerful when the ramp is a similar width to peak sensitivity SF (4cpd)	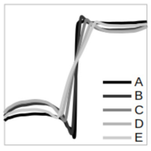	Similar to DoG (left)	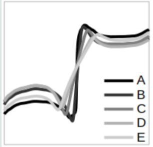
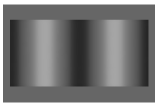	Hilbert-transformed Mach band	Various transforms have been shown to disrupt the Mach band effect, such as this Hilbert transform. These transforms generally simply remove the high spatial frequency “foot” of the Mach band.		Correctly predicts no Mach bands	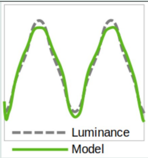	Correctly predicts no Mach bands	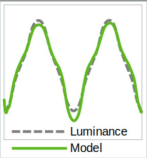
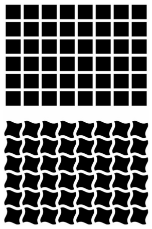	Hermann grid and wavy grid	The Hermann grid (upper image) causes dark spots to appear at the intersections between squares. The effect seems to depend on straight edges, and a curved grid (wavy grid, lower) does not create the illusory spots.	*[9]*	The DoG model does not simulate the effect. Altering the gain values enables the DoG model to simulate the effect, but then it is also present in the control wavy grid.	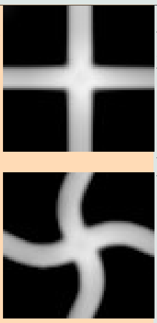	Correctly predicts that dark spots should appear on the straight-angled grid, but not with the wavy grid. The curved edges prevent the Gabor filters from bridging the gap between opposing corners.	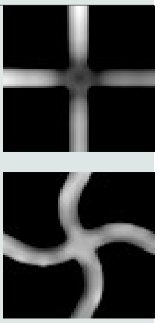
Contrast induction	A target’s internal contrast is influenced by the contrast of its surrounds. The causes are unclear, though are generally thought to depend on local normalisation of contrasts.						
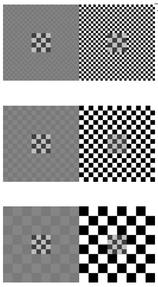	Textural contrast induction	Low contrast surrounds increase perceived target contrast, and this effect is most pronounced when the spatial frequency (SF) of the surround matches the target. In these example images the target on the left appears to have higher internal contrast than the same target on the right. The effect is most pronounced in the centre version with a matched spatial frequency.	*Adapted from [10]*	Target contrast is enhanced on a low-contrast background, and most powerfully for SF-matched background. Target SD is enhanced 4%, 17% and 11% for high SF, matched SF, and low SF respectively.	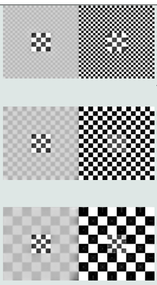	Correctly predicts effect more powerfully than DoG (left). The target SD is enhanced 19%, 24% and 22% for high SF, matched SF, and low SF respectively.	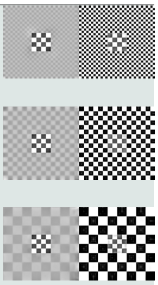

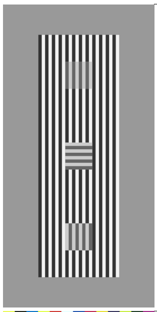	Orientation-dependent contrast induction ("tilt illusion")	High contrast surrounds reduce perceived target contrast when texture orientations match. In the example here the upper target has bars aligned with the background (in phase). In the centre is the same target rotated 90 degrees (orientations mismatched), and it appears to have a higher contrast. We also include a final control where the aligned target is out of phase with the surround. This target also appears to have higher contrast than the in-phase upper target (implying the effect is not entirely controlled by orientation).	*Custom figure with control, see [3] for similar effect.*	Interestingly the DoG model (without orientation sensitivity) is able to simulate the effect, albeit weakly. Compared to the top, internal SD is 6% higher in the middle target, and 4% higher in the lower target.	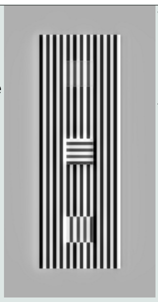	The oriented model is able to predict the contrast induction effect. Compared to the top, internal SD is 10% higher in the middle target and 11% higher in the lower target.	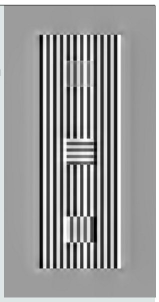
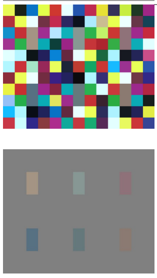	Chromatic contrast induction	High chromatic-contrast surrounds reduce perceived chromaticity. The high and low-contrast surrounds have the same luminance, red-green, and blue-yellow background averages. The targets appear to be more colourful (higher chromaticity) in the lower image.	*Adapted from [11]*	Chromaticity (average Euclidean distance from each target's colour to the background average) is 19% higher on the low contrast background.	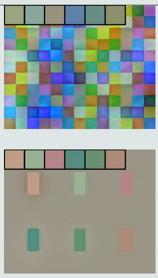	Chromatic channels use DoG, so only luminance varies (same 19% chromatic induction effect as left). The model also predicts chromatic grating induction in the high contrast surround.	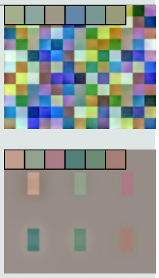

Colour constancy and chromatic adaptation

Colour constancy causes surfaces to appear to have the same colour under different lighting colours, generally attributed to chromatic adaptation. The mechanism by which this occurs is poorly understood, and models of whole scene averages, local surround averages and local maxima do not explain the effects fully [21].

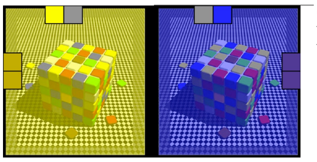	Lotto, Purves & Nundy cube	The cube is rendered with different simulated lighting conditions; yellow-tinted and blue-tinted. Colour-constancy causes grey tiles to appear blue in the yellow-tinted example, and yellow in the blue-tinted example.	*[12]*	Models colour constancy effects (i.e. grey in the left becomes blue, grey on the right becomes yellow). Also models brightness induction effect. 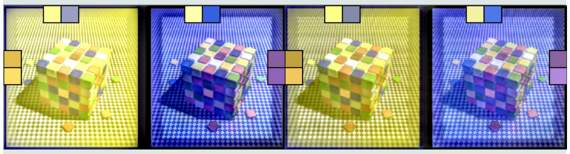			
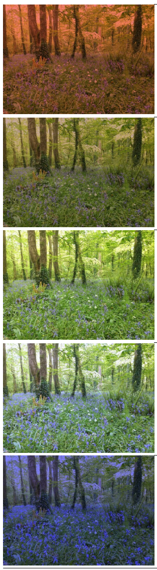	Simulated chromatic adaptation of natural scene, here the linear red channel is multiplied by 5<br>Red channel multiplied by 1.5<br>Neutral image<br>Blue channel multiplied by 3<br>Blue channel multiplied by 10	Chromatic adaptation lets us (and other animals) estimate the colour of an object even as the colour of the illuminant shifts. So, for example, as illuminant colour alters with weather and time of day, objects appear to stay the same colour. The capacity for maintaining colour constancy through chromatic adaptation is limited at some point by saturation levels.	*Generated example*	Chromatic modelling only uses DoG, however in this case we use the Gabor model for luminance.		The model is largely robust against even comparatively extreme differences in a scene's simulated illumination colour. Nevertheless, the model will start to show differences when the colours become so extreme that they saturate some spatial frequencies more. e.g. here the lower image has more blue-yellow saturation. Another interesting feature of the model is that it does not result in scene normalisation – this green scene of a woodland is predicted to be green by the model (not average grey)	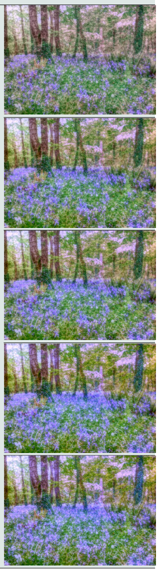

Chromatic simultaneous contrast

Simultaneous contrast causes a target's colour to shift in the opposite direction as its surrounds. This was one of the first visual illusions to have been described 1000 years ago by Ibn al-Haytham [23], who noted that green paint surrounded by blue appeared red-tinted, while the same paint surrounded by yellow appeared green-tinted.

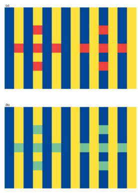	Chromatic simultaneous contrast	This example from Fairchild [13] shows a blue-yellow grating. The red squares (upper image) all have the same colour, and the blue squares (lower image) all have the same colour. Simultaneous contrast causes the upper left red to shift to blue/purple, and the upper right red to shift to yellow/orange. Likewise the lower left blue shifts to darker blue and the lower right shifts to pale green.	*[13]*		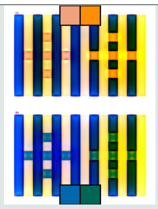		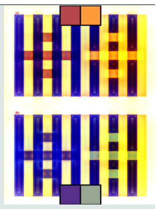

Colour Assimilation	Also known as the von Bezold spreading effect, this causes a colour to blend with the colour of its surrounds under certain circumstances. This is the opposite of simultaneous contrast, and early research established the conditions that cause each situation.

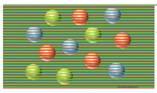	Spreading example with 3D spheres	This illusion developed by David Novick places beige spheres behind a colour grating (all these spheres are the same colour). Spreading causes dramatic colour shifts in the spheres depending on the colour of grating in front of them, making them appear red, green or blue.	*David Novick*		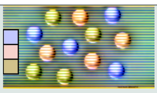		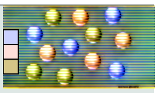
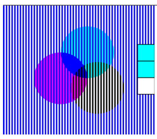	Subtractive colour circles illusion	This illusion places a cyan and magenta circle above a blue-white grating. The third circle is white, however simultaneous contrast makes it appear yellow. Spreading combines with the simultaneous contrast to make the intersection between cyan and white appear green.	*Generated*		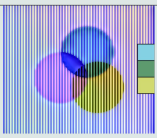		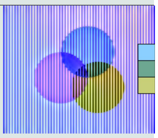
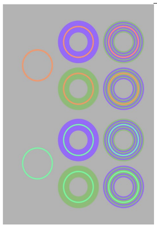	Monnier & Shevell illusion	Colour assimilation is found to be more powerful (i.e. colour blending with its surrounds more powerfully) with a striped surround than with a solid surround. In this example the orange ring is identical in all five upper instances, however the spreading effect is more powerful for the ring surrounded by stripes, than the rings surrounded by the same solid colours.	*Adapted from [14]*	Both models demonstrate powerful spreading effects, however they predict it should be more powerful with a solid surround.	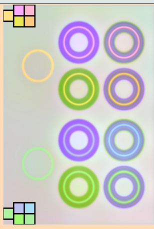	When adjusting the model to give higher spatial frequencies a higher gain, this effect can be modelled correctly.	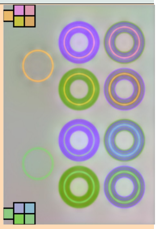

Colour Illusions	A number of the brightness illusions above are also powerful in a chromatic context (though not all). Interesting exceptions include illusory spots such as the Hermann grid (which our model suggests requires orientation-sensitive filters.

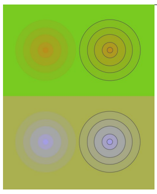	Chromatic Chevreul staircase	The concentric circles on the left appear to have internal gradients, but they are actually uniform flat colours. The black line surrounding the circles on the right eliminates the effect.	*Adapted from [15] & [22]*	The model is able to simulate the gradients in the staircase, and the control does show flat steps (although the effect reduces toward the centre)	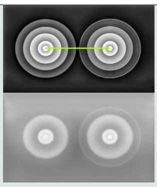	The output figure here shows the RG signal, processed with a bandwidth of 5	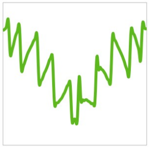
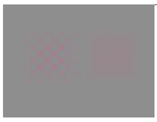	Patterns increase perceived saturation	Shapley et al. [22] show that a checker pattern (left) is perceived to have a higher saturation than the same colour averaged over a larger area (right), even though both have the same average cone stimulation.		We simulated Shapley et al.'s [22] data by multiplying the input image's RG signal by different values (graph's x-axis). The output RG signal for the checker pattern increases more than the area-averaged RG value (y-axis).			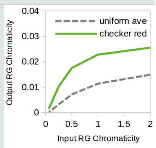

References:

1. Whittle P. Brightness, discriminability and the "Crispening Effect." Vision Research. 1992;32: 1493–1507. doi:10.1016/0042-6989(92)90205-W

2. Blakeslee B, McCourt ME. A unified theory of brightness contrast and assimilation incorporating oriented multiscale spatial filtering and contrast normalization. Vision Research. 2004;44: 2483–2503. doi:10.1016/j.visres.2004.05.015

3. Bertalmío M, Calatroni L, Franceschi V, Franceschiello B, Gomez Villa A, Prandi D. Visual illusions via neural dynamics: Wilson–Cowan-type models and the efficient representation principle. Journal of Neurophysiology. 2020;123: 1606–1618. doi:10.1152/jn.00488.2019

4. Geier J, Hudák M. Changing the Chevreul Illusion by a Background Luminance Ramp: Lateral Inhibition Fails at Its Traditional Stronghold - A Psychophysical Refutation. PLOS ONE. 2011;6: e26062. doi:10.1371/journal.pone.0026062

5. Gilchrist A. A Gestalt Account of Lightness Illusions. Perception. 2014;43: 881–895. doi:10.1068/p7751

6. Spehar B, Clifford CWG. The Wedding Cake Illusion: Interaction of Geometric and Photometric Factors in Induced Contrast and Assimilation. The Oxford Compendium of Visual Illusions. New York: Oxford University Press; 2017. doi:10.1093/acprof:oso/9780199794607.003.0059

7. Zaidi Q, Spehar B, Shy M. Induced Effects of Backgrounds and Foregrounds in Three-Dimensional Configurations: The Role of T-Junctions. Perception. 1997;26: 395–408. doi:10.1068/p260395

8. Kingdom FAA. Mach bands explained by response normalization. Frontiers in Human Neuroscience. 2014;8: 843. doi:10.3389/fnhum.2014.00843

9. Geier J, Bernáth L, Hudák M, Séra L. Straightness as the Main Factor of the Hermann Grid Illusion. Perception. 2008;37: 651–665. doi:10.1068/p5622

10. Chubb C, Sperling G, Solomon JA. Texture interactions determine perceived contrast. PNAS. 1989;86: 9631–9635. doi:10.1073/pnas.86.23.9631

11. Brown RO, MacLeod DIA. Color appearance depends on the variance of surround colors. Current Biology. 1997;7: 844–849. doi:10.1016/S0960-9822(06)00372-1

12. Purves D, Lotto RB, Nundy S. Why We See What We Do. Scientific American. 2002;90: 236–243.

13. Fairchild MD. Color appearance models. John Wiley & Sons; 2013.

14. Monnier P, Shevell SK. Large shifts in color appearance from patterned chromatic backgrounds. Nat Neurosci. 2003;6: 801–802. doi:10.1038/nn1099

15. Shapley R, Nunez V, Gordon J. Cortical double-opponent cells and human color perception. Current Opinion in Behavioral Sciences. 2019;30: 1–7. doi:10.1016/j.cobeha.2019.04.001

16. Solomon JA. The history of dipper functions. Attention, Perception & Psychophysics. 2009;71: 435–443. doi:10.3758/APP.71.3.435

17. Kane D, Bertalmío M. A reevaluation of Whittle (1986, 1992) reveals the link between detection thresholds, discrimination thresholds, and brightness perception. Journal of Vision. 2019;19: 16–16. doi:10.1167/19.1.16

18. Eagleman DM. Visual illusions and neurobiology. Nature Reviews Neuroscience. 2001;2: 920–926. doi:10.1038/35104092

19. Blakeslee B, Cope D, McCourt ME. The Oriented Difference of Gaussians (ODOG) Model of Brightness Perception: Overview and Executable Mathematica Notebooks. Behav Res Methods. 2016;48: 306–312. doi:10.3758/s13428-015-0573-4

20. Adelson EH. Lightness Perception and Lightness Illusions. 2nd ed. The new cognitive neurosciences. 2nd ed. MIT Press, Cambridge, MA; 2000.

21. Kraft JM, Brainard DH. Mechanisms of color constancy under nearly natural viewing. PNAS. 1999;96: 307–312. doi:10.1073/pnas.96.1.307

22. Shapley R, Nunez V, Gordon J. Cortical double-opponent cells and human color perception. Current Opinion in Behavioral Sciences. 2019;30: 1–7. doi:10.1016/j.cobeha.2019.04.001

23. Sabra AI. The Optics of Ibn al-Haytham: Books I-III On Direct Vision, 2 vols. 1989.
