# Supplementary material for: A model of colour appearance based on efficient coding of natural images
Source: PLoS Comput Biol. 2023 Jun 15;19(6):e1011117. doi: 10.1371/journal.pcbi.1011117 (PMC10270630; doi:10.1371/journal.pcbi.1011117)
Supplement: S1 Supporting Information — (PDF) [file pcbi.1011117.s002.pdf]

Supporting Information for: A model of colour appearance based on efficient coding of natural images

Jolyon Troscianko*[1] & Daniel Osorio[2]

[1] Centre for Ecology & Conservation, University of Exeter

[2] School of Life Sciences, University of Sussex

Corresponding author: jt@jolyon.co.uk

Description of the Spatiochromatic Bandwidth Limited model

Model input Requirements:

- A linear cone-catch image of known angular width. For example, cone-catch images created by the micaToolbox [1], or sRGB images converted to linear CIE XYZ channels. Our implementation accepts either sRGB images or cone catch images, and uses 32-bit images and processing throughout; 8-bits per channel is an insufficient dynamic range for coding linear natural scenes. The image should be scaled so that its resolution matches or exceeds the highest spatial frequency being modelled. For example, the DoG kernel we use has its peak wavelength sensitivity at 5.7 pixels, and the highest SF we model is 16 cpd, so the image should be scaled so that each degree of angular width has 16 x 5.7 pixels, i.e. 91.2 pixels per degree.
- Contrast sensitivity functions (CSFs) for the luminance and chromatic opponent channels (red-green and blue-yellow). Our code uses values from Kim et al. [2]. These values should be scaled so that contrasts are Michelson Contrast values - e.g. (red-green)/(red+green). Note that sensitivity is the inverse of the threshold contrast (i.e. *higher* sensitivity = *lower* threshold contrasts, Fig 2a in main text).
- Bandwidth values (ε) for luminance and each chromatic opponent channel (i.e. three values for human vision). These can be estimated from behavioural data (e.g. crispening effect, Fig 3a in main text), or from neurophysiological data (Fig 3b in main text). Suitable data are currently lacking for chromatic channel bandwidth, but we assume the red-green channel bandwidth equals that for the luminance channel, and the blue-yellow channel has about 30% of this bandwidth, in order to achieve efficient coding in natural scenes.

- Gain functions specify how each spatial frequency should be scaled following the clipping process. These are calculated by processing a library of images of natural scenes through the model with all gain values set to 1 (i.e. no gain), and measuring the resulting standard deviation of each channel. Normalising to these values gives output contrasts with standard deviations of 1 at each spatial frequency.

Spatial Frequency (cpd)	Luminance CSF	Luminance DoG gain	Luminance Gabor gain	Red-Green CSF	Red-Green gain	Blue-Yellow CSF	Blue-Yellow gain
0.125	10.885	0.201	0.0191	326.34	0.022	32.76	0.03
0.25	19.99	0.221	0.0181	326.34	0.023	32.76	0.035
0.5	43.07	0.179	0.0129	391.83	0.021	40.18	0.041
1	98.915	0.097	0.0066	370.11	0.023	42.2	0.046
2	133.32	0.074	0.0052	269.88	0.03	45.34	0.048
4	114.835	0.087	0.0062	187.68	0.038	38.69	0.054
8	90.825	0.109	0.008	92.72	0.049	14	0.065
16	18.955	0.309	0.0232				

Table A. Table of parameters. The contrast sensitivity functions (CSFs) here have been taking from Kim et al. [2], and converted to Michelson contrast. The gain functions are calculated by processing a bank of images of natural scenes using the model and measuring the standard deviation of contrasts at each spatial frequency.

Image Pre-Processing:

The cone catch image is converted to three channels: luminance, red-green opponency and blue-yellow opponency. The luminance channel is the average of all cone catch values from each receptor class, weighted by their cone ratios:

$$lum = 0.629R + 0.314G + 0.057B$$

Where R, G, and B are the longwave, mediumwave and shortwave cone catch pixel values respectively. Cone ratios here are from Hofer *et al.* [3].

The chromatic signals are calculated as Michelson contrasts:

$$RedGreen = \frac{R - G}{R + G}$$

Spatial Filtering

Each channel is convolved with either a Difference-of-Gaussian kernel or Gabor kernel. DoG kernels are orientation-insensitive, and are used for luminance and chromatic channels. Gabor kernels are orientation sensitive and are optionally used instead of DoG for the luminance channel. Our implementation uses conventional kernel functions (see code for exact parameters, examples shown in Fig 1b in main text); for the DoG the surround has a sigma value 1.6 times larger than the centre, and for the Gabor filter we use 4 orientations (sigma = 2, gamma = 1, frequency = 3). Our spatial filtering differs from that used previously in that we use Michelson Contrasts. Conventionally, the spatial filtering procedure uses logged input images and then applies a convolution. The result is mathematically identical to dividing the centre response by the surround. While this is computationally efficient, the resulting contrasts are asymmetric and unbounded (with a range of -1 to +infinity), and cannot be reliably matched to the behaviour described in CSFs because they are dimensionless ratios; see [4] for further discussion. The Michelson contrast output, φ, is on a scale of -1 to 1, where for example, a red spot with green surround that matches the kernel function would give a value of 1, the inverse arrangement would be -1, and grey (achromatic) would be 0. In the achromatic channel a light spot with dark surround would give 1, and the inverse would give -1. The chromatic channels have already had the Michelson contrast function applied, so the convolution is equivalent to simulating Michelson contrasts based on red-centre versus green-surround, or yellow-centre versus blue-surround giving contrast values. However, the Michelson contrast stage must be applied to the luminance channel following spatial filtering in order to compare centre and surround (or positive and negative regions in the Gabor kernel), i.e.:

$$\varphi = m \frac{centre - surround}{centre + surround}$$

Our implementation achieves this by calculating both signed and unsigned (absolute) convolutions for the numerator and denominator respectively. This differs slightly from Peli's approach, where the Gaussian pass-filtered luminance from a spatial frequency one octave lower was used as the denominator [4], but will give similar results. *m* is a parameter that scales the kernel's (arbitrary) amplitude to create contrasts that match the same scale as the contrast sensitivity functions. CSFs are generally calculated using sinewave gratings, so to calculate *m* we first create an image with a sinewave spatial frequency that matches the kernel's peak sensitivity (5.7 pixels in our case). The sinewave amplitude is set to a known Michelson contrast of e.g. 0.1, and then is convolved with the kernel. *m* is then the maximum contrast from the convolved image divided by the Michelson contrast of the input sinewave (i.e. 0.1). This scales the contrasts, φ, so that they are directly comparable to the conditions used to measure CSFs; *m* is 2.75, and 1.50 for the DoG and Gabor models respectively when using z-scored kernels (mean=0, SD=1).

Clipping

The activation threshold, α, is the inverse of contrast sensitivity, specified by the CSF at each spatial frequency, ϖ (see Fig Ba and table A for CSFs):

$$\alpha_{\varpi} = \frac{1}{ContrastSensitivity_{\varpi}}$$

Any contrasts below the saturation threshold are set to zero, while all other contrasts have the saturation threshold subtracted [5]:

$$If \varphi < \alpha_{\varpi} \text{ and } \varphi > 0, \varphi_{clipped} = 0$$
$$else if \varphi > 0, \varphi_{clipped} = \varphi - \alpha_{\varpi}$$

The sign is preserved for negative contrasts (i.e. the model assumes both centre-on and centre-off behaviour, described by positive or negative convolved pixel values respectively). However, in natural scenes the luminance DoG convolution results in negative contrasts that are twice as large as the positive ones (see Fig A). This does not apply to chromatic DoG or Gabor convolutions, where positive contrasts are symmetrical to negative contrasts.

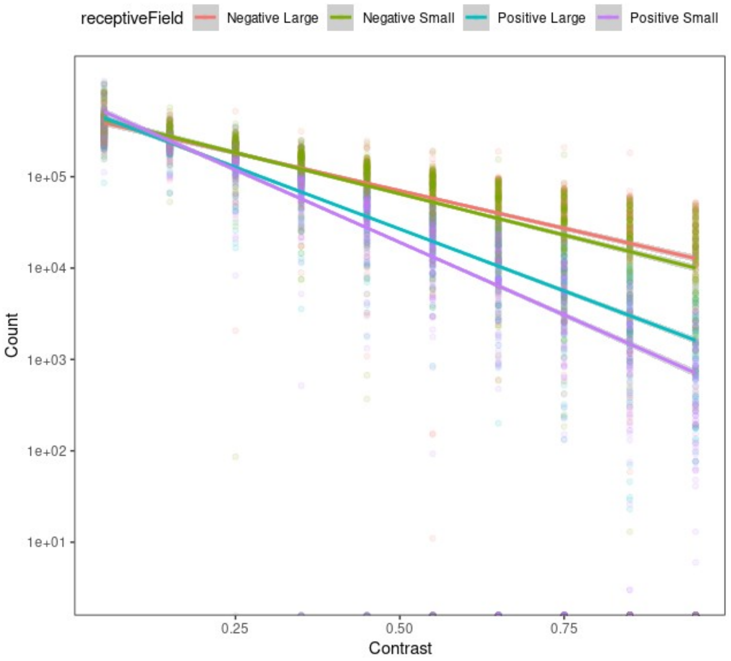

Figure A. Plot showing the DoG contrast (φ) before clipping is applied for both positive and negative contrasts in a bank of natural scene images with either conventional or enlarged DoG surrounds (“small” and “large”, with surrounds 1.6 and 1.92 times larger than the centre respectively, matching the 20% larger receptive field found in primate center-ON LGNs; [6]. The y-axis shows the logged count (number of pixels) in each image, corresponding to the contrast levels shown on the x-axis. The plot shows that negative contrasts are larger in a natural scene than are positive contrasts. Larger surrounds in the positive pathway reduces this contrast asymmetry slightly.

Following the principles of efficient coding, we assume that centre-on (positive) and centre-off (negative) DoG channels should use their bandwidth to code for the same dynamic range, i.e. both

positive and negative contrasts should have similar activation thresholds and saturation thresholds relative to the contrasts found in a natural scene. To achieve this in the DoG model we multiply α for negative contrasts by 2 i.e.:

$$If\varphi > -2\alpha_{\varpi} \text{ and } \varphi < 0, \varphi_{clipped} = 0$$
$$elseif\varphi < 0, \varphi_{clipped} = \varphi + 2\alpha_{\varpi}$$

Bandwidth, ε, is assumed to be uniform across all spatial frequencies, and this is used to calculate the saturation threshold, β, at each spatial frequency:

$$\beta_{\varpi} = \alpha_{\varpi}\varepsilon$$

The bandwidth can either be estimated by fitting the model to behavioural data (Fig 3a in main text), or based on the dynamic range of single neurones (Fig 3b in main text). Contrasts greater than the saturation threshold are set to equal the saturation threshold, creating a hard upper threshold. As above, negative contrasts are doubled for luminance DoG models (but not chromatic or Gabor models):

$$If\varphi > \beta_{\varpi}, \varphi_{clipped} = \beta_{\varpi}$$
$$elseif\varphi \leftarrow 2\beta_{\varpi}, \varphi_{clipped} = 2\beta_{\varpi}$$

This clipping process defines the dynamic range of the model at each spatial frequency. The result is that spatial frequencies with high contrast sensitivity also saturate much faster with increasing contrast, resulting in small dynamic range. Meanwhile spatial frequencies with low contrast sensitivity have a much larger dynamic range (Fig 2b in main text). However, the overlap in sensitivity between adjacent spatial frequencies means that almost all contrasts are within the dynamic range of one or more spatial frequencies (the orange areas in Fig 2f in main text), implying low bandwidths can be combined with high contrast sensitivity for efficient coding as long as there is a large range in dynamic ranges, and sufficient overlap in adjacent spatial frequencies. This explains why humans can perceive contrasts in natural scenes (or on high-definition televisions) over a dynamic range greater than 10,000:1, while our dynamic range for sinewaves is around 200:1. Fig B shows the proportion of a natural visual scene that undergoes clipping at different spatial frequencies.

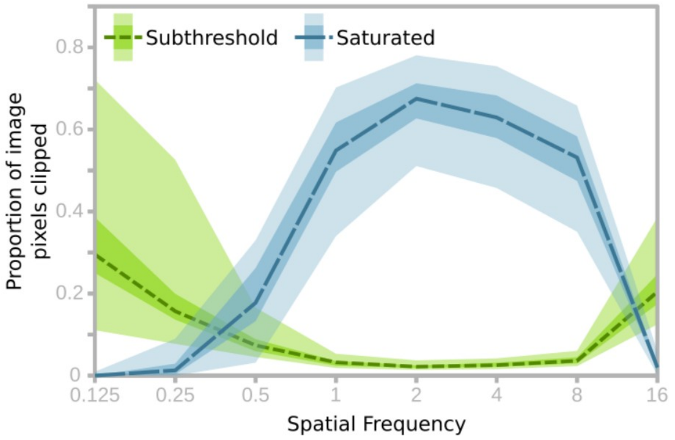

Figure B. Plot showing the proportion of pixels in each channel that are either saturated or sub-threshold in typical natural scenes. The results are based on 34 images of natural scenes, dashed lines show the median value, shaded areas show the interquartile range and full range of the data. High contrast sensitivity at intermediate spatial frequencies causes substantially more saturation, while the lower sensitivity channels show a substantial proportion of subthreshold contrasts.

Gain

Following clipping, contrasts are multiplied so that each spatial frequency results in equal contribution to the contrasts in the pooled image. i.e. in natural scene statistics each spatial frequency should contain equal contrast/information [7], however the clipping process substantially reduces the average amplitude of contrasts at intermediate spatial frequencies. The gain step equalises the average contrast amplitudes at each spatial frequency, i.e.:

$$\Phi = \frac{\varphi_{clipped}}{\sigma_{\varpi}}$$

Where σ_{ϖ} is the standard deviation of all $\varphi_{clipped}$ values in an image of a natural scene filtered at spatial frequency ϖ, resulting in gain-corrected contrasts, Φ.

Post-clipping smoothing

The hard upper and lower clipping thresholds (α and β) produce undesirable artefacts in the pooled image. We remove these by applying a Gaussian blur to each channel prior to pooling, with sigma values well below the filter’s spatial frequency (e.g. sigma value below 1 pixel radius, where the kernel’s peak wavelength sensitivity is 5.7 pixels). This step removes the artefacts, and the smoothing effect is responsible for the curvature near the saturation threshold shown in Fig 3b (in main text), matching the behaviour of primate ganglia [8]. This stage mirrors the correlated firing of neighbouring retinal ganglion cells cells where on-centre cells excite neighbouring on-centre cells, and likewise for off-centre cells, while on-centre and off-centre cells inhibit one-another [9].

Pooling

Pooling simply sums the contrast at each pixel location across each spatial frequency:

$$PooledOutput = \sum_{\varpi_{min}}^{\varpi_{max}} \Phi$$

This results in recombined luminance, red-green and blue-yellow chromatic channels. This output is designed to match subjective colour appearance, and it is therefore not straightforward to present these images on an sRGB display without confounding the very effects it seeks to predict. Nevertheless, we can convert back to a space that roughly approximates the cone-catch input:

$$R_{output} = \frac{2\,Lum}{1 + (1 - RedGreen)/(1 + RedGeen)}$$

$$G_{output} = \frac{R_{output}}{(1 + RedGreen)/(1 - RedGeen)}$$

$$B_{out\ put} = Lum \frac{1 + (1 - BlueYellow)}{1 + BlueYellow} - Lum$$

Supplementary References

1. Troscianko J, Stevens M. Image calibration and analysis toolbox – a free software suite for objectively measuring reflectance, colour and pattern. Methods Ecol Evol. 2015;6: 1320–1331. doi:10.1111/2041-210X.12439

2. Kim KJ, Mantiuk R, Lee KH. Measurements of achromatic and chromatic contrast sensitivity functions for an extended range of adaptation luminance. Human vision and electronic imaging XVIII. International Society for Optics and Photonics; 2013. p. 86511A.

3. Hofer H, Carroll J, Neitz J, Neitz M, Williams DR. Organization of the human trichromatic cone mosaic. The Journal of Neuroscience. 2005;25: 9669–9679.

4. Peli E. Contrast in complex images. J Opt Soc Am A. 1990;7: 2032. doi:10.1364/JOSAA.7.002032

5. Kulikowski JJ. Effective contrast constancy and linearity of contrast sensation. Vision research. 1976;16: 1419–1431.

6. Chichilnisky EJ, Kalmar RS. Functional Asymmetries in ON and OFF Ganglion Cells of Primate Retina. J Neurosci. 2002;22: 2737–2747. doi:10.1523/JNEUROSCI.22-07-02737.2002

7. Field DJ. Relations between the statistics of natural images and the response properties of cortical cells. Josa a. 1987;4: 2379–2394.

8. Derrington AM, Lennie P. Spatial and temporal contrast sensitivities of neurones in lateral geniculate nucleus of macaque. J Physiol. 1984;357: 219–240.

9. Nelson R. Visual Responses of Ganglion Cells. In: Kolb H, Fernandez E, Nelson R, editors. Webvision: The Organization of the Retina and Visual System. Salt Lake City (UT): University of Utah Health Sciences Center; 1995. Available: http://www.ncbi.nlm.nih.gov/books/NBK11550/
