# Supplementary material for: A model of colour appearance based on efficient coding of natural images
Source: PLoS Comput Biol. 2023 Jun 15;19(6):e1011117. doi: 10.1371/journal.pcbi.1011117 (PMC10270630; doi:10.1371/journal.pcbi.1011117)
Supplement: Attachment — Submitted filename: Review of PCOMPBIOL-D-22-0561_062622.pdf [file pcbi.1011117.s003.pdf]

Review of PCOMPBIOL-D-22-0561

Summary and overall evaluation

The authors present a novel low-level filtering model of color appearance based on the principle of efficient coding of natural images and they demonstrate that the model can account for a host of color phenomena, including some effects that are have often been ascribed to high- or mid-level processing. The paper is clearly written and the results are sound. The paper would make an important contribution to the literature on color appearance, but I have two main criticisms of the manuscript that I think need to be addressed.

First, there are a number of problems with the figures. In Figure 1(b), the filters are pixelated. That is okay as long as the illustration accurately represents the filter scale relative to the image pixel size. But you employ filters at different scales. Are you illustrating the finest scale here? This is not clear. In Figure 1(c), I believe you have the low and high spatial frequency labels reversed. If not, I don't know how to interpret this figure. Please address this in a revision. Figure 1(e) makes sense to include, but I am not sure how it informs the reader. Is the mismatch with the input of any noteworthy significance? Figure 2 seems like a mess to me. The aliasing at high spatial frequencies is distracting and I am not sure if it occurred in the actual modeling or if it is simply an artifact of the way in which you displayed the images on your monitor (as the authors suggest on lines 153-154). You really should eliminate these problems before publishing.

Second, I agree that the model can account for many important color phenomena and that this is noteworthy (and worth publishing). However, one might argue that a fairer evaluation of the model would emphasize what it cannot account for in addition to what it can. For example, it has been argued that the usual interpretation of the Chevreul illusion as a product of low-level filtering cannot explain the spatial extent of brightness induction from edges (see Geier & Hudak, PLoS ONE, 2011). This criticism appears relevant to the present model. Also, higher-level models based on atmospherics and assumptions about the illumination have been proposed for good reason. I don't accept that replacing a high- or mid-level model with a low-level model is more "parsimonious" without performing critical experiments that the reader could easily judge to definitively discriminate between the alternative hypotheses, since it is clearly possible that effects exist at multiple levels and it would be a mistake in that case to try to sweep the higher levels under the rug. Loose arguments based on parsimony are too perfunctory, despite the important results that have been achieved here.

At several points within the manuscript, the authors mention Retinex—another well-known approach to modeling color appearance. On lines 242-243, they specifically mention that the correct relative weightings of local and global factors in Retinex is unclear. In this context, they should take a look at (and reference) the following paper: M. E. Rudd, "Neurocomputational lightness model explains the appearance of real surfaces viewed under Gelb illumination," J. Percept. Imag., 3(1), 2020: DOI: 10.2352/J.Percept.Imaging.2020.3.1.010502. This paper directly estimates these relative weightings and combines them with assumptions about the relative neural gains associated with ON-center and OFF-center cells in the early visual system to arrive at a lightness appearance model that is shown to perform well against Gilchrist's lightness anchoring theory (whose work they refer to tangentially). Furthermore, the model is pitted against anchoring theory the context of psychophysical experiments that have been consider critical by Gilchrist himself for distinguishing lightness appearance models. The Rudd model is arguably superior to Retinex per se and it is—incidentally—another example of a feedforward model that does not require loops.

Minor points noted while reading the manuscript

Fig. 1(d)	I think you have the high and low spatial frequency labels reversed.
Fig. 2	The aliasing effects at high spatial frequency are very distracting. Can you avoid them?
line 176	I doubt that this difference would be statistically significant.
