# Supplementary material for: A model of colour appearance based on efficient coding of natural images
Source: PLoS Comput Biol. 2023 Jun 15;19(6):e1011117. doi: 10.1371/journal.pcbi.1011117 (PMC10270630; doi:10.1371/journal.pcbi.1011117)
Supplement: Attachment — Submitted filename: TrosciankoOsorioReview.pdf [file pcbi.1011117.s004.pdf]

There is a lot to like in this paper. There is a careful elaboration of similar existing models by incorporating circular and elongated filters, early nonlinearities, and chromatic as well as achromatic filters, all with some reference to published measurements. The model is demonstrated on a very large number of phenomena where the perceived color or brightness is shifted by spatial arrangements from what it would be in isolation. The paper would be a much more useful contribution if its components were based on more recent physiological measurements, and the predictions were quantitative not just qualitative, which is low-lying fruit that can be explained by many models. Below are my suggestions. These suggestions are limited by what I know best, so the authors could search the literature for more published retinal and cortical electrophysiology and for psychophysics that quantitatively takes apart many of the phenomena that they model.

Recent measurements of retinal ganglion cell properties show a diversity of cone combinations and spatial organization that need to be considered in any biologically plausible filter model, e.g. Wool, L. E., Packer, O. S., Zaidi, Q., & Dacey, D. M. (2019). Connectomic identification and three-dimensional color tuning of S-OFF midget ganglion cells in the primate retina. Journal of Neuroscience, 39(40), 7893-7909, and Wool, L. E., Crook, J. D., Troy, J. B., Packer, O. S., Zaidi, Q., & Dacey, D. M. (2018). Nonselective wiring accounts for red-green opponency in midget ganglion cells of the primate retina. Journal of Neuroscience, 1688-17.

The non-linearities in the model need to address recently measured differences in response compression between ON and OFF cells, e.g. Kremkow, J, Jin, J, Komban, S, Wang, Y, Lashgari, R, Li, X, Jansen, M, Zaidi, Q, Alonso, J M. Neuronal nonlinearity explains greater visual spatial resolution for darks than lights. Proceedings of the National Academy of Sciences 111.8 (2014): 3170-3175, and S J Komban, J Kremkow, Q Zaidi, Alonso, J M. Neuronal and perceptual differences in the temporal processing of darks and lights. Neuron, Volume 82, Issue 1, 2 April 2014, Pages 224–234 (2014).

Since the model includes chromatic filters that are different from achromatic filters in structure and spatial frequency, it would be good to see if it can predict quantitative differences between brightness and color induction, e.g. when induction is measured by keeping adaptation constant, brightness induction is more powerful than chromatic induction (Krauskopf, J., Zaidi, Q. and Mandler, M.B. Mechanisms of simultaneous color induction. J. Opt. Soc. Am., A3, 1752-1757, 1986), and brightness induction is spatially additive while color induction is attenuated by high frequency components (Zaidi, Q., Yoshimi, B., Flanigan, N. and Canova, A. Lateral interactions within color mechanisms in simultaneous induced contrast. Vision Research, 32, 1695-1707, 1992, also see DeBonet, J.S. and Zaidi, Q. Comparison between spatial interactions in perceived contrast and perceived brightness. Vision Research, 37, 1141-1155, 1997). The most comprehensive measurements of spatial additivity in brightness induction and the effect of local adaptation can be found in Spehar, B., DeBonet, J.S. and Zaidi, Q. Brightness induction from uniform and complex surrounds: a general model. Vision Research, 36, 1893-1906, 1996, and results for summing around the circumference of the test in Zaidi, Q. and Zipser, N Induced contrast from radial patterns. Vision Research, 33, 1281-1286, 1993, and Zaidi, Q., Yoshimi, B. and Flanigan, J. The influence of shape and perimeter-

length on induced color contrast. J. Opt. Soc. Am., A8, 1810-1817, 1991. In addition, the authors should test if their model can account for color induction in non-cardinal directions which it is claimed cannot be explained by induction along just the cardinal directions (Krauskopf, J., Zaidi, Q. and Mandler, M.B. Mechanisms of simultaneous color induction. J. Opt. Soc. Am., A3, 1752-1757, 1986).

The model's results for White's effect are surprising in not taking T-junctions into account, but a proper test would be to compare configurations that show the effects of T-junctions irrespective of higher-level 3D percepts (Zaidi, Q., Spehar, B. and Shy, M. Induced effects of backgrounds and foregrounds in three-dimensional configurations: the role of T junctions. Perception, 26, 395-408, 1997)

The claim is made that photoreceptor adaptation is too slow for the phenomena addressed in the paper, but this is unlikely to be true (see the references and analyses in Zaidi, Q, Ennis, R., Cao, D. and Lee, B. Neural locus of color afterimages. Current Biology, 22(3), 220 - 224, 2012.). Electrophysiological measurements show that edge effects that would be important in crispening and the Chevruel staircase require miniature eye-movements (Ennis, R, Lee, B and Zaidi, Q, Eye-movements and the neural basis of context effects on visual sensitivity. The Journal of Neuroscience, (2014), 34(24): 8119-8129), so it would be good to understand why the filters suffice without eye-movements and whether that is biologically plausible.

As I said, I am sure there are other quantitative papers that should also be addressed. I think the paper needs to make quantitative predictions to be a real advance over existing models to justify publication. I will be happy to review a revised version of the paper.
