# Supplementary material for: A model of colour appearance based on efficient coding of natural images
Source: PLoS Comput Biol. 2023 Jun 15;19(6):e1011117. doi: 10.1371/journal.pcbi.1011117 (PMC10270630; doi:10.1371/journal.pcbi.1011117)
Supplement: Attachment — Submitted filename: responses.pdf [file pcbi.1011117.s005.pdf]

We would like to thank all three reviewers for their constructive comments. We are delighted that the reviewers are generally highly positive, and in response to their suggestions we have made substantive edits to the manuscript, together with a considerable amount of additional analysis work. Please see our responses below in blue. Line numbers below refer to the document with track-changes shown.

Editor:

As you will see, the reviews are rather mixed. There is, however, some consensus that the work is mostly solid, but in the present form lacks the clear testable predictions that will give it sufficient novelty and biological relevance to be publishable in PLoS Computational Biology. Nonetheless, I decided to give you the opportunity to submit a substantially revised version that addresses the reviewers' concerns. In such cases it can occassionally happen that after a second round of reviews the manuscript is considered technically fine, but not of sufficient biological relevance for our journal. In such cases we frequently offer a direct transfer to PLoS One, where it then can be published often without an additional round of reviews. Of course, in this case, the decision whether you want to go this route will be with you as authors. This is not saying that this will be a likely outcome, but as I feel that this is a conceivable outcome given the current state, I wanted to communicate this possiblity clearly.

Many thanks for inviting resubmission. The comparatively simple nature of our model means that it is transparent and ideally placed to make testable predictions, particularly compared to mid or higher-level perceptual models. We show that our model fits neurophysiological data, psychophysical data, and over 50 phenomena that have otherwise been difficult to reconcile.

Reviewer #1: * General comments

- Asymmetry of ON and OFF channels

The doubling of the dynamic range of off-centre units introduces an important asymmetry (e.g., see Chichilnisky 2002). A discussion or quantification of how this asymmetry affects the results from the current model would be very interesting.

The reviewer points out that our model predicts the physiological asymmetries in ON vs OFF neural circuits, strengthening the model’s physiological relevance. We highlight (in lines 76-80 of supplementary material) the asymmetry between positive (centre-ON) and negative (centre-OFF) contrasts in natural scenes (for the model's DoG kernel) and draw attention to the neural asymmetry. We have provided further quantification of the magnitude of the effect (see supplementary figure 1), where we also demonstrate that enlarging the ON pathway's receptive

field by 20% (as is found in primate LGN ON cells) goes some way to reducing the asymmetry in contrasts in natural scenes. We also add a discussion of this asymmetry (in lines 285-293). While beyond the scope of our current study, we point out that (lines 292-293) it may be interesting to explore these asymmetries further, as they suggest that the neural asymmetries may reflect natural image statistics, rather than mechanistic limitations.

- Contrast mappings

A similar projective form for local contrast mapping was proposed by Peli (1990). Locally pooled activations also form the basis of divisive-normalisation approaches developed by Heeger and collaborators (see, e.g., 1992, 2020). Perhaps these studies could be cited and discussed.

We have now cited these useful models. Compared to Heeger's feedback-based divisive gain, our model assumes the gain functions are hard-wired to match natural scenes. Peli's model is indeed particularly relevant, and we now cite it widely. Peli's model measures contrast using Michelson equations (dividing the contrast by the luminance); this offers a valuable background framework for justifying our use of Michelson contrasts that we otherwise arrived at independently, so we are grateful for this suggestion. Our model builds on the Peli approach by adding the concept of limited bandwidth, and natural-image-statistic-based gain functions, and provides a perceptually uniform contrast output. We have mentioned the Heeger and Peli models in lines 113-115, 121-122.

- Feed-forward nature of the model

The model introduces considerable lateral interactions due to the divisive contrast mapping and gain equalisation. The authors could perhaps discuss how they interpret this quasi-instantaneous feedback loop. While technically implemented through a one-pass algorithm, this is not a strictly feed-forward system.

The model does not use divisive contrast mapping in the sense of Heeger models above. Hopefully our edits relating to the point above clarifies this and aids comparison; we assume gain is hard-wired at each spatial band. Thus, - it is a key feature of our model that the gain can be achieved at a neural level without any feedback. The only lateral pooling occurs in the local smoothing, which is primarily used to smooth the artefacts created by the hard activation and saturation thresholds. This is the opposite of divisive gain, and could also be achieved by sigmoidal (rather than hard) thresholds. However, we chose to keep hard thresholds for simplicity and generalisability.

- DoG and Gabor filters

In one of the two models, the authors use both DoG and Gabor filters (for chromatic and luminance filtering). Would this mean that both these representations exist at the same hierarchical level in visual processing - which is not likely given the organisation of the mammalian early visual system (although, see also Chauhan

2020)? It would be interesting to know how the authors interpret this design decision.

We would stress that our model does not seek to accurately model specific neural processes, rather it seeks to build a generalisable colour appearance model based on efficient use of limited bandwidth when processing natural scenes. Efficient coding would imply that reduction in bandwidth (or information capacity) should occur early in the system, and thereafter the information's dynamic range should be preserved. So, the principles of saturation and activation thresholds that our model lays out could occur at multiple hierarchical levels simultaneously. In support of this, we find a bandwidth of ~15:1 is required to match the DoG function to human behavioural data and LGN neural behaviour, whereas Gabor filters (at four angles) would only need a bandwidth of ~4:1, suggesting that cortical cells would require a lower bandwidth-per cell to transmit the same information, and would result in surprisingly similar clipping-based artefacts (i.e. the two models are strikingly similar qualitatively).

- Linearity of the Michelson mapping

line 349: It is not very clear how the chromatic Michelson contrasts can be considered to distribute over a convolution. The contrast is a nonlinear operation with respect to the raw cone-inputs. Or perhaps I have misunderstood the computation that is being performed?

The Michelson contrast calculation for both luminance and chromatic processing produces a number between -1 and 1, and is non-linear (though is reversible at this stage before clipping). This links with Peli's work that you point out above, although compared to Peli we base the contrast on the kernel's own absolute product, where Peli uses a Gaussian filter at a larger spatial scale. For the chromatic channels the Michelson contrast convolution could feed the red and green channels into the kernel separately, however for more efficient coding we use inputs that have already had the Michelson contrast applied at each pixel beforehand, so a conventional convolution can be used instead. Whichever is done the output is the same, so we chose to use the more computationally efficient approach. An achromatic grey patch will create a 0 output from the convolution, a red centre and green surround (matching the kernel perfectly) would give 1, and the reverse would give -1. We have added this description to the text and hope this helps clarify the process (lines 29-35 and 42-44 of supplementary material)

- Control experiments

To explore the role of various components of the model, perhaps the authors could employ control experiments. For instance, as suggested above, a very interesting manipulation would be to observe the effect of varying the degree of asymmetry between contrast increments and decrements. Another interesting manipulation is the use of other forms of contrast mapping such as Weber contrast.

This is a good suggestion. We have added extra modelling of the asymmetry in ON and OFF pathways in natural scenes for DoG contrasts (Supplementary figure 1). We have also added a simulation of the effects of contrast increments on targets with light or dark surrounds. This is an elegant way to show where simultaneous contrast is predicted to switch to the White illusion as contrast increases (see updated Figure 4).

We prefer not to use Weber contrast because the output is unbounded, which creates difficult asymmetries when working out the activation and saturation thresholds, and confusion when dealing with zeros. Peli's 1990 paper that you pointed out helps to justify this decision.

- Comparisons with other models and Degree of freedom

The authors could also compare their model to other cololur appearance models such as the CIECAM02. A particularly interesting quantity would be a list of free parameters for each of the experiments that were simulated. Since the model has clearly defined phsyical interpretations for each of its parameters, this could be very informative for psychophysicists designing new studies.

The CIECAM02 model does not adapt to local (within-image) luminance or contrast cues. So, for example, in our crispening effect test it matches the CIE L model, with slight variation based on viewing parameters that we change. However, these parameters refer to viewing conditions outside of/surrounding the image itself, there is no inflection point in the curve that shifts with image properties. As such we do not believe this would be a useful comparison.

Our model has no free parameters, and no alterations were made to the model throughout the demonstrations/experiments shown in the appendix (other than DoG versus Gabor variants). We agree that making the parameters we used more easily accessible would be valuable, and have added supplementary table 1. This contrasts with the CIECAM02 and CIECAM16 models, which have long lists of parameters.

* Manuscript related

- The description of the model in the main manuscript is not very clear. The authors provide a much more detailed description in the Supplementary Material which could perhaps be moved to the main text.

We will happily take editorial advice here. We believe that most readers would appreciate a simplified overview.

- Figure 2f: What do the various colours mean?

The colours here (and throughout the manuscript) show where subthreshold clipping or saturation affects pixel values. The key is at the top of this figure, and links with panels b and c.

- Figure 4 : The background colour is not indicated in the Lightness Difference plots.

Apologies for lack of clarity here. This plot shows the difference in lightness (contrast) between adjacent squares. The veridical values would all be the same as the squares increase in luminance at a fixed rate. This plot shows how the contrast difference for the three rows varies, matching the apparent effect.

- Line 253: The sentence is incomplete.

Fixed.

- lines 369-377: The equations are not very clear. The symbol \phi_{clipped} has not been defined before.

Michelson contrast (phi) was described (on line 351 of the original manuscript file), but we agree could have been clearer. We have adjusted the description, which links with a question above concerning clarity of the Michelson contrast calculation (lines 29-35 of supplementary material).

- line 376: It is not very clear what the authors mean by efficient coding here.

We have re-written this section and hope it is clearer (lines 76-80 of supplementary material).

- line 347: Why is a nonlinear contrast mapping not mappable to CSFs? CSFs are nonlinear.

We have re-written this section, which links to the above comments regarding Peli's (1990) use of Michelson contrasts. Hopefully this is clearer now (lines 29-35 in supplementary material)

Reviewer #2: Michael E. Rudd

Summary and overall evaluation

The authors present a novel low-level filtering model of color appearance based on the principle of efficient coding of natural images and they demonstrate that the model can account for a host of color phenomena, including some effects that are have often been ascribed to high- or mid-level processing. The paper is clearly written and the results are sound. The paper would make an important contribution to the literature on color appearance, but I have two main criticisms of the manuscript that I think need to be addressed.

We are delighted to have received such a positive appraisal.

First, there are a number of problems with the figures. In Figure 1(b), the filters are pixelated. That is okay as long as the illustration accurately represents the filter scale relative to the image pixel size. But you employ filters at different scales. Are you illustrating the finest scale here? This is not clear.

Our implementation works entirely in the spatial domain (not via Fourier transform), and in order to achieve computational efficiency it scales down the image repeatedly in octave steps and uses the same kernel at each scale. Therefore, this pixelation accurately reflects the model's operation. Nevertheless, there is an interpolation stage that smooths these pixelations out, and we have updated the figure to include this instead.

In Figure 1(c), I believe you have the low and high spatial frequency labels reversed. If not, I don't know how to interpret this figure. Please address this in a revision.

Indeed, this figure was incorrectly labelled and is now fixed, many thanks for pointing it out.

Figure 1(e) makes sense to include, but I am not sure how it informs the reader. Is the mismatch with the input of any noteworthy significance?

This image highlights some concepts that we feel are valuable. For example, this image has had the dynamic range compressed in much the same way as an impressionist painting or tone-mapped HDR photograph. The details in the shaded parts of the image are amplified dramatically. Another noteworthy feature is the dramatic over-representation of the bluebell flowers. When walking around a bluebell wood people often describe a carpet of blue in person, and then complain that photos cannot do it justice. A further point is that this image of a woodland scene is predominantly green, and would remain green to our model even if the input image's white balance were altered. We have altered the image caption to briefly highlight some of these points.

Figure 2 seems like a mess to me. The aliasing at high spatial frequencies is distracting and I am not sure if it occurred in the actual modeling or if it is simply an artifact of the way in which you displayed the images on your monitor (as the authors suggest on lines 153-154). You really should eliminate these problems before publishing.

The input image's spatial frequency in this demonstration was matched to the kernels peak spatial frequency (so aliasing was not present in the modelling behind this figure, though there were minor harmonic effects that are to be expected, and these can be seen in the secondary peaks in figure 2e and 2f). We have re-made this figure and have reduced aliasing patterns by increasing the resolution, and artificially blurring the higher spatial frequencies in the input and output images.

We believe that this image is valuable for demonstrating how a system with small bandwidth of 15 levels that has a peak contrast sensitivity of ~200:1 can make us believe we are seeing contrasts in a natural scene that are 10,000:1 or higher. Some of these concepts are difficult to convey, and these figures have been received well in talks explaining the model, but we would be open to other suggestions for how to convey the model's bandwidth versus dynamic range behaviour visually.

Second, I agree that the model can account for many important color phenomena and that this is noteworthy (and worth publishing). However, one might argue that a

fairer evaluation of the model would emphasize what it cannot account for in addition to what it can. For example, it has been argued that the usual interpretation of the Chevreul illusion as a product of low-level filtering cannot explain the spatial extent of brightness induction from edges (see Geier & Hudak, PLoS ONE, 2011). This criticism appears relevant to the present model.

We agree, and have added discussion of Geier and Hudák’s study (lines 235-245).

Also, higher-level models based on atmospherics and assumptions about the illumination have been proposed for good reason. I don’t accept that replacing a high- or mid-level model with a low-level model is more “parsimonious” without performing critical experiments that the reader could easily judge to definitively discriminate between the alternative hypotheses, since it is clearly possible that effects exist at multiple levels and it would be a mistake in that case to try to sweep the higher levels under the rug. Loose arguments based on parsimony are too perfunctory, despite the important results that have been achieved here.

We do not seek to dismiss higher-level models, only to develop a usable colour appearance model that matches behavioural data. If we have misrepresented any models or concepts then we would happily address them.

Nevertheless, if two models have the same explanatory power, the “simpler” one is to be preferred, so the question is: i) whether the different models do all have the same explanatory power, and ii) whether our model is more “simple” than others. We believe that we have extensive evidence for point (i) as we have tested more phenomena in a single study than any other models we are aware of. The results and source code are all made available to the reader. Point (ii) (model complexity) could be judged with inferred minimum numbers of neural layers to simulate the model's effects, and here we believe our model could be implemented in a single layer, while (true to their names) the mid and higher-level models are necessarily more complex. The fact that our model would need to be compared to multiple others (haze, anchoring etc...) adds further weight to our suggestion of parsimony. However, we agree with the reviewer that more quantitative data would be highly valuable for testing our model against others. This would be beyond the scope of this study, but we mention it in lines 244-245.

At several points within the manuscript, the authors mention Retinex—another well-known approach to modeling color appearance. On lines 242-243, they specifically mention that the correct relative weightings of local and global factors in Retinex is unclear. In this context, they should take a look at (and reference) the following paper: M. E. Rudd, “Neurocomputational lightness model explains the appearance of real surfaces viewed under Gelb illumination,” J. Percept. Imag., 3(1), 2020: DOI: 10.2352/J.Percept.Imaging.2020.3.1.010502. This paper directly estimates these relative weightings and combines them with assumptions about the relative neural gains associated with ON-center and OFF- center cells in the early visual system to arrive at a lightness appearance model that is shown to perform well against Gilchrist’s lightness anchoring theory (whose work they refer to tangentially). Furthermore, the model is pitted against anchoring theory the context of psychophysical experiments that have been consider critical by Gilchrist himself for

distinguishing lightness appearance models. The Rudd model is arguably superior to Retinex per se and it is—incidentally—another example of a feedforward model that does not require loops.

This is indeed a relevant study to cite, and we now do so (lines 219-221), and we also cite an earlier Rudd and Zemach study (line 224). Together with reviewer 1's comments regarding on versus off centre pathways it would be interesting to investigate this further, particularly in light of asymmetries in natural scene statistics. The performance of our Gabor-based model is also noteworthy though, as this does not have any asymmetry (in natural scene statistics or in our model), and performs remarkably similarly to the DoG model.

Minor points noted while reading the manuscript Fig. 1(d) I think you have the high and low spatial frequency labels reversed.

Fixed

Fig. 2 The aliasing effects at high spatial frequency are very distracting. Can you avoid them?

Reviewer 1 also mentions this, and we have attempted to fix the issue, thanks for highlighting it.

line 176 I doubt that this difference would be statistically significant.

Indeed, a t-test of the residuals of these two models is non-significant and we add this comparison (lines 177-178).

Reviewer #3: Qasim Zaidi

There is a lot to like in this paper. There is a careful elaboration of similar existing models by incorporating circular and elongated filters, early nonlinearities, and chromatic as well as achromatic filters, all with some reference to published measurements. The model is demonstrated on a very large number of phenomena where the perceived color or brightness is shifted by spatial arrangements from what it would be in isolation.

Many thanks for the positive comments!

The paper would be a much more useful contribution if its components were based on more recent physiological measurements, and the predictions were quantitative not just qualitative, which is low-lying fruit that can be explained by many models. Below are my suggestions. These suggestions are limited by what I know best, so the authors could search the literature for more published retinal and cortical electrophysiology and for psychophysics that quantitatively takes apart many of the phenomena that they model.

As mentioned above, our goal was not to model neurophysiological pathways exactly, but to create a simple (and plausibly generalisable) perceptually uniform colour appearance model. Indeed, it is striking that our model performs so well

given its comparative simplicity, e.g. linear receptor responses, hard thresholds, symmetric ON and OFF pathways, no free parameters, and the parameters it does use can all be determined through behavioural data. This last point is key for developing a “generalisable” model that can be developed and tested in a wide range of non-humans.

Therefore, while we accept that future avenues of research could refine the model by adding complexity, this would entail considerable research that is well beyond the aims and scope of this study.

We do offer two lines of quantitative model testing, for both top-down (crispening) and bottom-up (primate LGN responses). We believe the fact that our model reconciles both, generating the same saturation threshold, is noteworthy. Few other experimental datasets were found to have suitable data for quantitative comparisons.

Recent measurements of retinal ganglion cell properties show a diversity of cone combinations and spatial organization that need to be considered in any biologically plausible filter model, e.g. Wool, L. E., Packer, O. S., Zaidi, Q., & Dacey, D. M. (2019). Connectomic identification and three-dimensional color tuning of S-OFF midget ganglion cells in the primate retina. Journal of Neuroscience, 39(40), 7893-7909, and Wool, L. E., Crook, J. D., Troy, J. B., Packer, O. S., Zaidi, Q., & Dacey, D. M. (2018). Nonselective wiring accounts for red-green opponency in midget ganglion cells of the primate retina. Journal of Neuroscience, 1688-17.

Many thanks for these suggestions, however as noted above, our model does not seek to simulate any particular hierarchical level within the visual pathway. For example, our Gabor-model (which performs in similar ways to the DoG model) is closer to cortical pathways.

The non-linearities in the model need to address recently measured differences in response compression between ON and OFF cells, e.g. Kremkow, J, Jin, J, Komban, S, Wang, Y, Lashgari, R, Li, X, Jansen, M, Zaidi, Q, Alonso, J M. Neuronal nonlinearity explains greater visual spatial resolution for darks than lights. Proceedings of the National Academy of Sciences 111.8 (2014): 3170-3175, and S J Komban, J Kremkow, Q Zaidi, Alonso, J M. Neuronal and perceptual differences in the temporal processing of darks and lights. Neuron, Volume 82, Issue 1, 2 April 2014, Pages 224–234 (2014).

Indeed, this links nicely with reviewers 1 and 2 above, who also mention these asymmetries. We have added citations and a discussion of this asymmetry (lines 285-293). We would point out, however, that the Gabor model performs similarly without any asymmetric modelling.

Since the model includes chromatic filters that are different from achromatic filters in structure and spatial frequency, it would be good to see if it can predict quantitative differences between brightness and color induction, e.g. when induction is measured by keeping adaptation constant, brightness induction is more powerful than chromatic induction (Krauskopf, J., Zaidi, Q. and Mandler, M.B.

Mechanisms of simultaneous color induction. J. Opt. Soc. Am., A3, 1752-1757, 1986), and brightness induction is spatially additive while color induction is attenuated by high frequency components (Zaidi, Q., Yoshimi, B., Flanigan, N. and Canova, A. Lateral interactions within color mechanisms in simultaneous induced contrast. Vision Research, 32, 1695-1707, 1992, also see DeBonet, J.S. and Zaidi, Q. Comparison between spatial interactions in perceived contrast and perceived brightness. Vision Research, 37, 1141- 1155, 1997). The most comprehensive measurements of spatial additivity in brightness induction and the effect of local adaptation can be found in Spehar, B., DeBonet, J.S. and Zaidi, Q. Brightness induction from uniform and complex surrounds: a general model. Vision Research, 36, 1893-1906, 1996, and results for summing around the circumference of the test in Zaidi, Q. and Zipser, N Induced contrast from radial patterns. Vision Research, 33, 1281- 1286, 1993, and Zaidi, Q., Yoshimi, B. and Flanigan, J. The influence of shape and perimeter- length on induced color contrast. J. Opt. Soc. Am., A8, 1810-1817, 1991. In addition, the authors should test if their model can account for color induction in non-cardinal directions which it is claimed cannot be explained by induction along just the cardinal directions (Krauskopf, J., Zaidi, Q. and Mandler, M.B. Mechanisms of simultaneous color induction. J. Opt. Soc. Am., A3, 1752-1757, 1986).

Quantitative comparisons are generally not straightforward. For example, the papers mentioned here quantify the effects using two individuals whose performance is not - and would not be expected to be - identical. This sample size means population variance cannot be estimated. Therefore, were we to attempt direct quantitative comparisons (which would each take considerable time), if our model predictions didn't fall exactly between the two human observer's values we would effectively be unable to assess the model’s performance. We would also point out that - as we mention in the manuscript - our model's chromatic parameters are far less well understood, and require future behavioural work. So direct comparisons between quantitative chromatic and achromatic performance may be premature.

The model’s results for White’s effect are surprising in not taking T-junctions into account, but a proper test would be to compare configurations that show the effects of T-junctions irrespective of higher-level 3D percepts (Zaidi, Q., Spehar, B. and Shy, M. Induced effects of backgrounds and foregrounds in three-dimensional configurations: the role of T junctions. Perception, 26, 395-408, 1997)

The extensive examples provided in Zaidi et al. (1997) are indeed very useful additions, particularly given the well described viewing conditions (which we can replicate), and so we have added them to our study. The Gabor model generally predicts the direction of the effects well, although the DoG model does not perform quite so well (which we discuss in lines 193-194 & 241-244).

The claim is made that photoreceptor adaptation is too slow for the phenomena addressed in the paper, but this is unlikely to be true (see the references and analyses in Zaidi, Q, Ennis, R., Cao, D. and Lee, B. Neural locus of color afterimages. Current Biology, 22(3), 220 - 224, 2012.). Electrophysiological measurements show that edge effects that would be important in crispening and the Chevruel staircase

require miniature eye-movements (Ennis, R, Lee, B and Zaidi, Q, Eye-movements and the neural basis of context effects on visual sensitivity. The Journal of Neuroscience, (2014), 34(24): 8119-8129), so it would be good to understand why the filters suffice without eye-movements and whether that is biologically plausible.

We accept that all neurones have both spatial and temporal receptive fields, and our model is simplistic in that – like existing colour appearance models – it primarily considers the spatial dimension. However, we note that our bandwidth limit likely reflects temporal neural constraints, and that the principles of coding efficiency apply in both space and time.

The phenomena we test appear to generate illusory effects extremely quickly (e.g. they appear when you saccade to- and away-from the images as fast as possible). Given human saccades are typically just a few hundred milliseconds, the afterimage-based work (adaptation over many seconds) above does not seem highly relevant to our point. The eye-movement based work likewise is presenting an unnatural control where eye movements are effectively prevented. Interestingly the illusions we test appear to remain after fixating on them without eye movements for a long time. In any case, the instantaneous effects described in the eye-movement based study are likely the ones we test.

As I said, I am sure there are other quantitative papers that should also be addressed. I think the paper needs to make quantitative predictions to be a real advance over existing models to justify publication. I will be happy to review a revised version of the paper.

We believe that our perceptually uniform colour appearance model shows that limited bandwidth (which must occur in early visual processing) can at least partially explain a host of phenomena that were previously thought to require much more complex explanations. We have also systematically tested more phenomena than any other study we are aware of, matching both neurophysiological and psychophysical quantitative data. This reviewer therefore sets an exceptionally high bar that is at odds with reviewers 1 and 2. Nevertheless, we hope that our edits and inclusion of their own studies helps demonstrate the value of our model.

Have the authors made all data and (if applicable) computational code underlying the findings in their manuscript fully available?

The PLOS Data policy requires authors to make all data and code underlying the findings described in their manuscript fully available without restriction, with rare exception (please refer to the Data Availability Statement in the manuscript PDF file). The data and code should be provided as part of the manuscript or its supporting information, or deposited to a public repository. For example, in addition to summary statistics, the data points behind means, medians and variance measures should be available. If there are restrictions on publicly sharing data or code —e.g. participant privacy or use of data from a third party—those must be specified.

Reviewer #1: Yes

Reviewer #2: Yes

Reviewer #3: **No:**

The source code for our model was included in the original submission, so the reviewers should feel able to test the code themselves.
