# Supplementary material for: A model of colour appearance based on efficient coding of natural images
Source: PLoS Comput Biol. 2023 Jun 15;19(6):e1011117. doi: 10.1371/journal.pcbi.1011117 (PMC10270630; doi:10.1371/journal.pcbi.1011117)
Supplement: Attachment — Submitted filename: Review of Trosianko & Osario, PLOS Comp Biol_012323.pdf [file pcbi.1011117.s006.pdf]

Review

The authors have satisfied all of my earlier criticisms. This is a nice paper, which makes a unique and important contribution to the field of color and lightness perception by arguing that a number of well-known "illusions" can be understood in terms of efficient coding. I am personally not convinced that mid-level processes play no role in these phenomena, but it is important to show how far we can get based on various reasonable assumptions about the nature of low-level mechanisms, and the authors have done that here.

Figures

In my previous review, I was critical of the figures. The authors have done a good job of cleaning them up

Lesser points

214-216 This is not really a sentence. You could place a comma before it and write it as a continuation of the previous sentence, though that would be a long sentence.

219 You should also cite the following recent paper, which fleshes out this model in much more detail and—among other things—shows that the model predicts the Chevreul illusion in a strictly feedforward manner (unlike Geier and Hudak's model, which is based on a diffusive filling-in mechanism):

Rudd, M. E. (2022). A feedforward model of spatial lightness computation by the human visual system. *Electronic Imaging*, 34, 167-1 - 167-7, https://doi.org/10.2352/EI.2022.34.11.HVEI-167
