# Supplementary material for: A model of colour appearance based on efficient coding of natural images
Source: PLoS Comput Biol. 2023 Jun 15;19(6):e1011117. doi: 10.1371/journal.pcbi.1011117 (PMC10270630; doi:10.1371/journal.pcbi.1011117)
Supplement: Attachment — Submitted filename: responses Rev2.pdf [file pcbi.1011117.s007.pdf]

We are delighted that you are likely to accept our revised manuscript. The reviewers' comments were both minor, and we have made the small edits requested (highlighted in blue).

Reviewer #1: The authors have responded satisfactorily to most queries.

Many thanks for the positive appraisal.

To nitpick, I disagree that changing the order of the convolution and Michelson-contrast operation will give the same result. While it does so for the boundary conditions that the authors outline (a grey patch, or template-match inputs), the intermediate points will differ. However, the newer version of the manuscript does not raise this issue as the authors have described the exact computation they have implemented.

We are glad that the reviewer believes our current approach is open and clear.

Reviewer #2:

The authors have satisfied all of my earlier criticisms. This is a nice paper, which makes a unique and important contribution to the field of color and lightness perception by arguing that a number of well- known "illusions" can be understood in terms of efficient coding. I am personally not convinced that mid-level processes play no role in these phenomena, but it is important to show how far we can get based on various reasonable assumptions about the nature of low-level mechanisms, and the authors have done that here.

Many thanks; we agree that it is important to see how far generalised low-level assumptions can help to explain these phenomena.

Figures

In my previous review, I was critical of the figures. The authors have done a good job of cleaning them up

We agree that the figures are much improved – many thanks for the constructive suggestions.

Lesser points

214-216 This is not really a sentence. You could place a comma before it and write it as a continuation of the previous sentence, though that would be a long sentence.

We have re-written this sentence, which now reads: "Recent work has successfully modelled some lightness phenomena by simulating edge integration with asymmetric gain for centre-on and centre-off pathways (Rudd 2020, Rudd 2022), but the mechanisms underlying colour constancy are less clear (Kraft and Brainard 1999)."

219 You should also cite the following recent paper, which fleshes out this model in much more detail and—among other things—shows that the model predicts the Chevreul illusion in a strictly feedforward manner (unlike Geier and Hudak's model, which is based on a diffusive filling-in mechanism):

Rudd, M. E. (2022). A feedforward model of spatial lightness computation by the human visual system. Electronic Imaging, 34, 167-1 – 167- 7, https://doi.org/10.2352/EI.2022.34.11.HVEI-167

We now cite this useful recent paper – many thanks.
